# Study on the Relationship between Leisure Activity Participation and Wearing a Mask among Koreans during COVID-19 Crisis: Using TPB Model

**DOI:** 10.3390/ijerph17207674

**Published:** 2020-10-21

**Authors:** Young-Jae Kim, Jeong-hyung Cho, Seung-Woo Kang

**Affiliations:** Department of Physical Education, Chung-Ang University, Seoul 06974, Korea; yjkim@cau.ac.kr (Y.-J.K.); cheer1007@naver.com (J.-h.C.)

**Keywords:** COVID-19, Theory of Planned Behavior, wearing a mask, leisure, physical activity, non-physical activity

## Abstract

This study utilizes the Theory of Planned Behavior (TPB) variables—including “attitude,” “subjective norms,” and “perceived behavioral control”—to understand the relationship between mask-wearing behavior and physical/non-physical leisure activity participation in Koreans as well as the tendencies behind mask-wearing intentions within leisure activities. The measurement tools used attitude, subjective norms, control, and mask use intention factors based on the TPB. Overall, 545 individuals participated, and the non-overlapping regions, sex, and age were considered through the stratified sampling method. The survey was conducted online owing to COVID-19, and collected data were derived through descriptive and multiple linear regression analyses. First, a difference in mask-wearing intention based on physical and non-physical leisure activities was identified; second, attitudes and perceived behaviors were considered in light of the dangers posed by COVID-19. It was found that control influences the tendency of intention to wear a mask depending on whether the group was engaged in physical or non-physical activity. Therefore, it can be stated that mask-wearing must be mandatory during physical and non-physical activities owing to respiratory diseases such as COVID-19. It is also important to simultaneously promote a positive attitude toward mask-wearing to enable people to believe that they can stay in full control of their own health.

## 1. Introduction

Today, Corona Virus Disease-19 (COVID-19) has led to a global halt in the working and daily lives of people, and its prolonged impact has brought changes not only to society in general but also to leisure and social activities [1,2].

South Korea has campaigned for social distancing to prevent the spread of COVID-19 and additional cases at the national level (less than 50 confirmed cases in stage 1, 50 to less than 100 confirmed cases in stage 2, and more than 100–200 confirmed cases in stage 3). As the population resumes their lives under these new conditions, it is mandatory to wear masks. Infection prevention is ensured by checking body temperature when entering buildings, checking mask use, stopping public exercise, reducing the operation of high-risk facilities in the private sector, and compulsory compliance with facility quarantine regulations. Infection is controlled by mandatory mask use as well as temperature-taking and ensuring that masks are worn when entering buildings [3].

Mask-wearing behavior is being utilized as a top-priority practice in preventing infections and maintaining good health [4,5]. Therefore, interest in mask-wearing increased exponentially, regardless of age or sex. However, Feng et al. [3] found that some did not wear masks despite the consistent recommendations that symptom-showing individuals and those in the healthcare environment wear masks. In other words, there are complaints about the inconvenience of wearing masks at all times due to the COVID-19 situation [6,7,8]. Moreover, people complained about the inconvenience of wearing protective equipment in various national disaster scenarios (Middle East Respiratory Syndrome: MERS, Severe Acute Respiratory Syndrome: SARS, and swine flu) even before the COVID-19 crisis. These inconveniences were mainly related to the negative influence of cognitive, behavioral, and physical symptoms of mask-wearing daily and during leisure activities [9,10]. As such, the amount of physical activity has declined due to self-isolation and social distancing due to the COVID-19 outbreak, and the discomfort of mask-wearing influences physical activity [11].

Koreans have been engaging in physical activity while wearing masks and have spent a significant amount of time indoors due to social distancing. They are, thus, experiencing drastic changes in their daily lives in comparison to pre-COVID-19 days. However, physical activities are still essential despite the COVID-19 environment; while protecting against sources of infections through mask-wearing, leisure activities that are either physical or non-physical and can be carried out while maintaining social distancing are necessary [12,13]. Within this context, this study sought to utilize the Theory of Planned Behavior (TPB) by Ajzen [14] to verify mask-wearing intentions in physical and non-physical leisure activities.

Ajzen [14] developed the TPB to understand the psychological decision factors behind behavioral change. The factors proposed within the TPB are attitude, subjective norms, and perceived behavioral control. Among these, attitude is a determinant of behavioral change, and attitude toward behavior has long been considered an influential factor in forming behavior. This assumption has been supported by empirical evidence [15,16,17]. Generally, based on empirical evidence, it improves the intentions of the individual participating in a behavior, and develops an advantageous attitude toward a specific behavior. Next, subjective norms form people’s behavior through social influences, in addition to their personal attitudes. In particular, this factor can incentivize their behavior through their normative beliefs, which subsequently protect against social rejection and fear [18].

Furthermore, it has also been shown that people’s beliefs are influenced by social comparisons with those around them [19]. People tend to engage in behavior that is aligned with the subjective norms, which reflect others’ social expectations of the individual. Lastly, the perceived behavioral control factor is not only influenced by individual attitudes and social norms but is also formed through the evaluation of the ability to perform a specific behavior. Similar to self-efficacy in the social cognitive theory proposed by Bandura [20], perceived behavioral control is an individual’s assessment of their own ability to carry out the behavior at hand [14].

In other words, if a person wears a mask based on their own judgment and can control this particular action, their intention toward this behavior will increase. Moreover, there is significant empirical evidence on the effectiveness of TPB in explaining an individual’s intentions to wear masks [21,22]. Mask-wearing intentions can be explained through a summary of the above situation and in accordance with Guillaumie et al. [23], and TPB [24,25] can be used to verify mask-wearing behavioral intentions through the most effective factors relating to behavioral execution. Therefore, the purpose of this study is to use Ajzen’s [14] TPB to investigate attitudes toward mask use, subjective norms, and perceived behavioral control in order to discover the mask-wearing intentions of people during participation in leisure activities in the COVID-19 situation. The focus is on analyzing the data that this study is expected to provide for safe participation in leisure activities.

## 2. Materials and Methods

### 2.1. Sample and Participants

This study was conducted with the general public in their 20s to 50s participating in leisure activities amidst the COVID-19 situation in Korea. The survey was conducted online from 20 July to 25 July 2020 through Macromill Embrain, a Korean research firm. This study obtained informed consent from survey participants and based its research on mask-wearing behavior during physical and non-physical leisure activities during COVID-19. A total of 600 questionnaires were distributed for this study, with a recovery rate of 92.3%; in total, 545 responses were used for the final analysis after excluding 9 multiple (or omitted) or inaccurate responses. Participants were selected by region, sex, and age using the stratified sampling method. The sample sizes were classified as 100 = poor; 200 = moderate, which is the sample size used for each grade in Comrey and Lee’s quantitative study [26]; 300 = good; 500 = very good; and ≥1000 = excellent. Considering these classifications, a sample of 545 was used for this study. This study was conducted after receiving the approval of the Institutional Review Board of Chung-Ang University (1041078-202007-HRSB-172-01).

### 2.2. Measurements

This study used a cross-sectional method to evaluate the mask-wearing behaviors of Korean leisure activity participants in the COVID-19 situation. The survey scales included Attitude (AT, 3 items), Subjective Norms (SN, 3 items), Perceived Behavioral Control (PBC, 3 items), and Intention (INT, 2 items), which are factors in TPB [14,25], as well as 6 other items for demographic data including gender, age, marital status, monthly income, leisure activities (physical and non-physical), and mask-wearing. In terms of the mask-wearing item, this study utilized the standard mask-wearing method proposed by the Korea Centers for Disease Control (KCDC) [27] and created the survey with the options “mask worn during leisure activity,” “mask sometimes worn,” and “mask not worn” based on expert discussions with one medical expert and two leisure experts. These items are positive questions rated on a 5-point Likert scale ranging from “not at all” (1 point) to “very much” (5 points).

#### Theory of Planned Behavior

The TPB by Ajzen [14] used in this study was measured using 11 items modified for Korean circumstances [14,25] in order to understand the psychological determinants of behavioral change. These items were measured on 5-point Likert scales. Cronbach’s α of the original scale in the previous study was 0.90, while it was α = 0.64 in this study. To verify whether they were suitable for factor analysis and were normally distributed, this study examined the standard fit (Kaiser–Meyer–Olkin; KMO), which was found to be 0.78; Bartlett’s test of sphericity yielded a chi-squared approximation of 3632.16, *p* < 0.001. The varimax orthogonal rotation method was also used in this study. The total cumulative variance was found to be 82.39. In addition, the value of h^2^ was 0.83 to 0.90 for the SN factor, 0.78 to 0.83 for the PBC factor, 0.67 to 0.87 for the AT factor, and 0.85 to 0.87 for the INT factor Table 1.

### 2.3. Data Analysis

This study utilized the SPSS Window 25.0 (Version 25.0, IBM, Armonk, NY, USA) software. This study conducted a frequency analysis to understand demographic characteristics and a correlation analysis to verify the relationship between physical and non-physical leisure activities and mask-wearing behavior. In addition, a multiple linear regression analysis was conducted to confirm the influence of mask-wearing behavior on leisure activity type. To increase the reliability of the analysis, the significance level was set at *p* < 0.05 for analysis.

## 3. Results

### 3.1. Sociodemographic Characteristics of Korean Leisure Activity Participants

Table 2 shows mask-wearing behavior according to the characteristics of Korean leisure activity participants. First, in terms of differences in mask-wearing behavior between the genders, men (*N* = 274, 50.3%) reported subjective norms of 2.24 ± 0.99, whereas women (*N* = 271, 49.7%) reported the following: 2.83 ± 0.42 for attitude, 4.41 ± 0.59 for perceived behavioral control, and 3.73 ± 0.71 for intention; these values indicate that women generally had higher levels of attitude, perceived behavioral control, and intention factors. In terms of age, those in their 50s (*N* = 137, 25.1%) had higher levels of attitude (2.79 ± 0.39) and perceived behavioral control (4.39 ± 0.56), and those in their 30s (*N* = 136, 25.0%) had high levels of subjective norms (2.02 ± 1.06) and intentions (4.28 ± 0.68). In terms of marital status, the married group (*N* = 300, 55.0%) showed high levels of attitude (2.79 ± 0.43), subjective norms (2.07 ± 0.93), and intention (3.70 ± 0.70) in relation to mask-wearing behavior.

Table 3 shows the leisure activities that Koreans participated in during the COVID-19 situation. It shows the leisure activities of the participants of this study since the COVID-19 outbreak in January 2020. In terms of physical activity, they have been going on walks (*N* = 170, 31.2%), going to the gym (*N* = 109, 20%), and engaging in home training (*N* = 56, 10.3%). In terms of non-physical activity, they visited cafes (*N* = 71, 13.0%), read books (*N* = 62, 11.4%), and done arts and crafts (*N* = 47, 8.6%). Thus, some have continued to engage in leisure activities.

### 3.2. Sociodemographic Characteristics of Participants and Their Behavior of Wearing Masks during Leisure Activities

Table 4 shows mask-wearing activity based on the social characteristics of Korean leisure activity participants. First, in terms of the influence of perceived behavioral control, subjective norms, and attitudes of men and women on their mask-wearing behavior, men (*β* = 0.32, *p* < 0.00, 1.5%, R^2^ = 0.15) were more influenced by the attitude factor as compared to women (*β* = 0.26, *p* < 0.00, 1.3%, R^2^ = 0.13). However, women (*β* = 0.20, *p* < 0.00) were more influenced by perceived behavioral control as compared to men (*β* = 0.10, *p* < 0.11). Next, this study examined the influence of mask-wearing on the intention wearing masks during physical and non-physical leisure activities. First, in terms of physical activity, the attitude factor influenced the always-wearing group (*β* = 0.27, *p* < 0.00, 1.7%, R^2^ = 0.17), sometimes-wearing group (*β* = 0.26, *p* < 0.01, 0.7%, R^2^ = 0.07), and non-wearing group (*β* = 0.42, *p* < 0.01, 2.2%, R^2^ = 0.22) in a descending order. Among these groups, perceived behavioral control (*β* = 0.25, *p* < 0.00) influenced intention. In terms of non-physical activity, the attitude factor influenced the always-wearing group (*β* = 0.32, *p* < 0.00, 2.0%, R^2^ = 0.20), sometimes-wearing group (*β* = 0.29, *p* < 0.00, 1.4%, R^2^ = 0.14), and non-wearing group (*β* = 0.26, *p* < 0.00, 0.9%, R^2^ = 0.09) in a descending order. Perceived behavioral control only influenced mask-wearing behavior in the always-wearing group (*β* = 0.21, *p* < 0.01) and sometimes-wearing group (*β* = 0.18, *p* < 0.04). Lastly, in terms of mask-wearing behavior according to marital status, attitude (*β* = 0.20, *p* < 0.00, 0.9%, R^2^ = 0.09) and perceived behavioral control (*β* = 0.15, *p* < 0.03) influenced intention in single individuals, and attitude (*β* = 0.33, *p* < 0.00, 2.0%, R^2^ = 0.20), perceived behavioral control (*β* = 0.22, *p* < 0.00), and subjective norms (*β* = 0.13, *p* < 0.02) influenced intention in married individuals.

## 4. Discussion

This study analyzed the tendencies of mask-wearing intention during leisure activity based on the TPB model to underscore the differences in mask-wearing behavior according to the social characteristics of Korean participants and to confirm their mask-wearing intentions.

First, in terms of the demographic differences between leisure activity participants during the COVID-19 situation, attitude and perceived behavioral control were gender-specific mask-wearing behavioral factors that had significant influence. Nevertheless, it can be seen that both men and women wear masks for their own safety and health [28,29].

Perceived behavioral control influenced female leisure activity participants as a factor in mask-wearing intention, whereas attitude influenced male leisure activity participants. More specifically, this means that this factor had a significant influence on women’s mask-wearing intentions in any situation, regardless of obstacles. On the other hand, men decided to wear masks based on individual experiences and evaluations, which are heavily influenced by the attitude factor. Therefore, it is necessary to conduct further research on when men do and do not wear masks. If the rationale for not wearing masks is due to the inconvenience and discomfort of wearing masks, it is important to suggest measures to reduce such inconveniences to encourage them to wear masks. Furthermore, it would be necessary to emphasize that consistent mask-wearing is important for their health.

Second, there were differences in mask-wearing during leisure activities depending on the marital status of Korean participants, and attitude, subjective norms, and perceived behavioral control were factors that influenced mask-wearing intentions in the married group. This is in line with the study by Oseni, Agbede, Fatusin, and Odewale [30], which showed the influence of health as well as the psychological and social well-being of the family on the individual. Further, this appears to be in line with the attitude of maintaining a healthy lifestyle for the family that they need to protect, which subsequently influences their intentions to wear masks during leisure activities in order to protect the health of their significant others and their families. Therefore, Korea needs to formulate a social atmosphere where an individual’s own and their family’s health are being safeguarded through their determination and concerns for others.

Third, the tendency to wear masks in those who always wore masks throughout physical leisure activities were influenced by attitude and perceived behavioral control. This demonstrates the attitude of protecting one’s health from infection sources through mask-wearing as well as a positive attitude toward physical exercise. Mask-wearing intentions appear to be influenced by the attitude and control required in regular physical exercise [31,32,33,34,35,36]. In this context, Freeman and Eykelbosh [37] also indicate that people with mask-wearing intentions continue to engage in leisure activities amidst social distancing regulations by maintaining safety guidelines for physical exercise, and that they had a strong belief in mask wearing during physical activity. In addition, [38,39] other research indicates that people continue to engage in leisure activities amidst social distancing regulations by keeping safety guidelines for physical exercise, and that they had a strong perception of mask-wearing during physical activity.

Fourth, “attitude” and “perceived behavioral control” influenced mask-wearing intentions in groups that always wore masks and sometimes wore masks and engaged in non-physical leisure activities. Mask-wearing in non-physical activities appears to have been influenced by individual attitudes and perceived behavioral control through the recommendations on wearing masks during social activities. This is in line with the infection-prevention guidelines from governments. Additionally, the tendencies of mask-wearing intentions appear to have risen due to the risk of infection [40,41,42]. Several studies, for example the study by Yang, Li, Garg, and Qi [43] emphasize the importance of wearing masks and suggest that people should wear masks when engaging in daily and leisure activities because of the virus’ ability to spread rapidly. Koreans appear to be engaging in this behavior of effectively wearing masks by inducing behavior through the intention of wearing masks as a preventive, safety-enhancing activity during their physical and non-physical activities, influenced by factors such as government-imposed, strong social distancing policies, mask availability, and the social atmosphere.

The intention to wear a mask during physical and non-physical exercise had a significant influence on each group. However, in terms of physical activity, the perceived behavioral control factor influenced the always-wearing group and influenced the always-wearing and sometimes-wearing groups engaging in non-physical activity. In other words, wearing masks for non-physical activity in the present COVID-19 situation appears to be an important individual characteristic. Therefore, wearing masks throughout physical and non-physical activities is predicted by the mask-wearing attitude in terms of maintaining one’s own health.

## 5. Conclusions

This study aimed to confirm the psychological changes of Korean leisure activity participants wearing masks amid alterations in social conditions caused by COVID-19. As a result, the tendency of the intention to wear a mask based on physical and non-physical leisure activities was explained. Unlike other TPB studies, this study predicted the intention of mask use in modern individuals as a way to prevent infection during participation in leisure activities during COVID-19. In addition, it was confirmed that the activity of wearing a mask is perceived as promoting positive health behaviors during leisure activities. Given the dangers posed by COVID-19 today, attitudes and perceived behavioral controls influence the tendency of intention to wear a mask depending on the group that is engaging in physical or non-physical leisure activities. Accordingly, it is necessary to ensure that wearing a mask is mandated in light of respiratory diseases such as COVID-19, while promoting not only a positive attitude so that people can take full control of their health but also important safety information about public health. Alternatively, it is important to tailor the promotion of the mask-wearing behavior for people who engage in physical and non-physical activities outdoors and indoors through message training.

However, this study has some limitations. First, this study made causal inferences when using a cross-sectional data analysis, which should be avoided. Future studies should involve longitudinal surveys or experimental designs to clarify causal relationships. Specific measures are required to interpret the influence of physical activity on mask-wearing intention by educating people on the positives and negatives of masks in the COVID-19 situation. Second, this study investigated mask-wearing behavioral intentions during physical activity by using a one-dimensional structure. Future studies should conduct detailed research on the health problems of modern society by recreating this study and through comprehensive research on mask-wearing behavioral intentions during physical activity in terms of climate issues and private and public domains. Furthermore, future studies should test the effectiveness of TPB in environmental contexts, such as health-related problems as well as non-environmental contexts. Lastly, understanding how attitudes toward health behavior, social norms, and perceived behavioral control change based on health campaigns would be helpful in executing health promotion campaigns.

## Figures and Tables

**Table 1:** Exploratory factor analysis of Theory of Planned Behavior.

Item	Factor				h^2^
	1	2	3	4	
SN2	0.92	−0.24	−0.05	−0.01	0.90
SN1	0.90	−0.19	−0.06	−0.00	0.86
SN3	0.90	−0.14	0.01	−0.03	0.83
PBC2	−0.17	0.87	0.15	0.09	0.81
PBC3	−0.22	0.85	0.20	0.11	0.83
PBC1	−0.20	0.83	0.19	0.11	0.78
AT3	0.02	0.13	0.91	0.12	0.87
AT4	0.05	0.16	0.88	0.10	0.81
AT2	−0.22	0.28	0.71	0.19	0.67
INT3	0.03	0.10	0.09	0.92	0.87
INT2	−0.06	0.14	0.24	0.88	0.85
Reliability	0.92	0.88	0.84	0.83	
Eigenvalue	2.64	2.42	2.28	1.72	
Variance (%)	23.98	22.03	20.76	15.62	
Cumulative variance (%)	23.98	46.02	66.77	82.39	

Kaiser–Meyer–Olkin (KMO) = 0.78, χ^2^ = 3632.16, df = 55, *p* < 0.001, SN: subjective norms, PBC: perceived behavioral control, AT: attitude, INT: intention.

**Table 2:** Demographic characteristics of mask-wearing behavior in Koreans (*N* = 545).

Variables	*N* (%)	AT	SN	PBC	INT
		M ± SD	M ± SD	M ± SD	M ± SD
**Gender**					
Male	274 (50.3)	2.69 ± 0.46	2.24 ± 0.99	4.23 ± 0.64	3.65 ± 0.77
Female	271 (49.7)	2.83 ± 0.042	1.75 ± 0.84	4.41 ± 0.59	3.73 ± 0.71
Total	545				
**Age (years)**					
20s	135 (24.8)	2.74 ± 0.46	2.00 ± 0.98	4.36 ± 0.64	3.70 ± 0.80
30s	136 (25.0)	2.72 ± 0.50	2.02 ± 1.06	4.28 ± 0.68	3.76 ± 0.73
40s	137 (25.1)	2.77 ± 0.43	2.02 ± 0.95	4.25 ± 0.60	3.66 ± 0.66
50s	137 (25.1)	2.79 ± 0.39	1.92 ± 0.81	4.39 ± 0.56	3.64 ± 0.76
Total	545				
**Marital status**					
Single	242 (44.4)	2.72 ± 0.45	1.91 ± 0.97	4.34 ± 0.63	3.69 ± 0.77
Married	300 (55.0)	2.79 ± 0.43	2.07 ± 0.93	4.30 ± 0.62	3.70 ± 0.70
Bereaved	3 (0.6)	2.44 ± 1.02	1.33 ± 0.58	4.67 ± 0.58	2.67 ± 1.53
Total	545				

SN: subjective norms, PBC: perceived behavioral control, AT: attitude, INT: intention.

**Table 3:** Leisure activities of Koreans amidst COVID-19 (*N* = 545).

Leisure Type			
Physical Activity	*N* (%)	Non-Physical Activity	*N* (%)
Walking	170 (31.2)	Visiting cafes	71 (13.0)
Gym	109 (20)	Reading	62 (11.4)
Home training	56 (10.3)	Crafts	47 (8.6)
Running	37 (6.8)	Cooking	46 (8.4)
Cycling	33 (6.1)	Eating out	45 (8.3)
Yoga	30 (5.5)	Driving	39 (7.2)
Golf	22 (4.0)	Meeting people	38 (7.0)
Pilates	22 (4.0)	Resting	38 (7.0)
Swimming	18 (3.3)	Games	34 (6.2)
Badminton	16 (2.9)	Watching movies	34 (6.2)
Soccer	12 (2.2)	Drawing pictures	27 (5.0)
Basketball	9 (1.7)	Watching TV	23 (4.2)
Baseball	6 (1.1)	Listening to music	16 (2.9)
Tennis	5 (0.9)	Writing	14 (2.6)
		Musical instruments	11 (2.0)
Total	545	Total	545

**Table 4:** Regression analysis of mask-wearing behavior according to participants’ demographic characteristics and leisure activities (*N* = 545).

Category		IV	N-Std. C		Std. C	*p*	R^2^
			B	St. E	Β		
Gender	Male	PBC	0.18	0.09	0.15	0.03	0.15
		SN	0.08	0.05	0.10	0.11	
		AT	0.53	0.11	0.32	0.00	
	Female	PBC	0.24	0.08	0.20	0.00	0.13
		SN	0.01	0.05	0.01	0.89	
		AT	0.44	0.10	0.26	0.00	
Physical activity	Always worn	PBC	0.29	0.07	0.25	0.00	0.17
		SN	0.03	0.04	0.05	0.42	
		AT	0.45	0.10	0.27	0.00	
	Sometimes worn	PBC	0.11	0.10	0.09	0.29	0.07
		SN	0.09	0.06	0.12	0.12	
		AT	0.44	0.13	0.26	0.01	
	Not worn	PBC	0.15	0.19	0.12	0.13	0.22
		SN	−0.04	0.13	−0.05	0.43	
		AT	0.59	0.21	0.42	0.00	
Non-physical activity	Always worn	PBC	0.26	0.10	0.21	0.01	0.19
		SN	0.01	0.06	0.01	0.86	
		AT	0.50	0.12	0.32	0.00	
	Sometimes worn	PBC	0.20	0.10	0.18	0.04	0.14
		SN	0.05	0.06	0.06	0.45	
		AT	0.48	0.13	0.29	0.00	
	Not worn	PBC	0.16	0.10	0.13	0.13	0.09
		SN	0.09	0.06	0.11	0.14	
		AT	0.45	0.14	0.26	0.00	
Marital status	Single	PBC	0.19	0.09	0.15	0.03	0.09
		SN	−0.02	0.05	−0.02	0.77	
		AT	0.35	0.12	0.20	0.00	
	Married	PBC	0.25	0.07	0.22	0.00	0.20
		SN	0.10	0.04	0.13	0.02	
		AT	0.54	0.10	0.33	0.00	

IV: independent variables, N-Std. C: non-standardized coefficient, Std. C: standardized coefficient, Std. E: standard error, SN: subjective norms, PBC: perceived behavioral control, AT: attitude.
